# Undifferentiated Embryonal Sarcoma of the Liver: First Case Reported in Central America

**DOI:** 10.1002/cnr2.70067

**Published:** 2024-11-25

**Authors:** Amilcar Antonio Alfaro García, Ludwing Bacon Fonseca, Maria del Rosario Romero Alvarez

**Affiliations:** ^1^ Department of Hepatopancreatobiliary Surgery Manolo Morales Peralta Hospital Managua Nicaragua; ^2^ Department of Oncology Manolo Morales Peralta Hospital Managua Nicaragua; ^3^ Department of Pathology Vivian Pellas Hospital Managua Nicaragua

**Keywords:** cancer, case report, liver, undifferentiated embryonal sarcoma

## Abstract

**Background:**

Undifferentiated embryonal sarcoma of the liver (UESL) is a rare and aggressive mesenchymal tumor, typically occurring in children. It poses significant diagnostic challenges due to its atypical presentation and rarity in clinical practice.

**Case:**

We report the first case in Central America of a 19‐year‐old female presenting with chronic abdominal pain and a liver mass. Imaging revealed a solid and cystic lesion in the liver, and a subsequent complete surgical resection confirmed the diagnosis of UESL. The patient received adjuvant chemotherapy and has been disease‐free for 34 months.

**Conclusion:**

This case highlights the importance of complete surgical resection combined with chemotherapy to improve prognosis in UESL. Our findings emphasize the need to consider UESL in differential diagnoses of liver lesions, especially in regions where it has not been previously reported.

## 1 Introduction

Undifferentiated embryonal sarcoma of the liver (UESL) was first described in 1978 by Stocker et al. [1]. It is a rare and highly aggressive mesenchymal tumor. While UESL typically affects children, its occurrence in adults is exceedingly rare, making accurate diagnosis particularly difficult [2].

The rarity of UESL, especially in Central America, makes this case particularly noteworthy, as it highlights the need for awareness and consideration of UESL in the differential diagnosis of liver lesions. Accurate and early diagnosis is critical for initiating effective treatment, which can significantly improve survival rates. The etiology is unknown, but it is usually accompanied by genetic aberrations, including amplification or deletion in chromosomes 1q, 5p, 8p, and 12q, translocation of chromosome 19q13.4, and mutations in p53 [3].

Few cases have been described in adults, and its presentation in this group poses a diagnostic challenge. Generally, other benign pathologies that are more frequent, such as biliary cystadenoma or hydatid cyst, are excluded first. Imaging studies show a liver mass with a mixed component (solid and cystic), pseudocapsule, and areas of necrosis inside. However, there is no pathognomonic sign that guides the diagnosis of UESL [4]. Clinically, the liver mass may be accompanied by pain with or without fever, normal liver biochemical markers, and usually normal alpha‐fetoprotein (AFP) [5].

UESL is an aggressive malignant tumor with disease‐free survival of up to 35% at 5 years in some older series, although more recent studies show a disease‐free survival of around 80% and overall survival rates of 90% at 5 years in pediatric cases [6, 7, 8].

Current therapeutic approaches emphasize the importance of a multimodal strategy, which includes neoadjuvant chemotherapy followed by delayed complete surgical resection. Studies suggest that this combined treatment offers the best chance of disease‐free survival. The purpose of this report is to share the first documented case of UESL in Central America.

### 1.1 Case Presentation

This is a 19‐year‐old female patient with a history of chronic abdominal pain lasting one year, located in the right hypochondrium, and associated with non‐significant weight loss. The condition was initially managed at another center as a liver abscess. Percutaneous drainage of the lesion was attempted without success, leading to her referral to our unit. The case study was carried out at Manolo Morales Peralta Hospital, Nicaragua, from January 2021 to December 2023.

### 1.2 Patient Information and Clinical Findings

**FIGURE 1:**
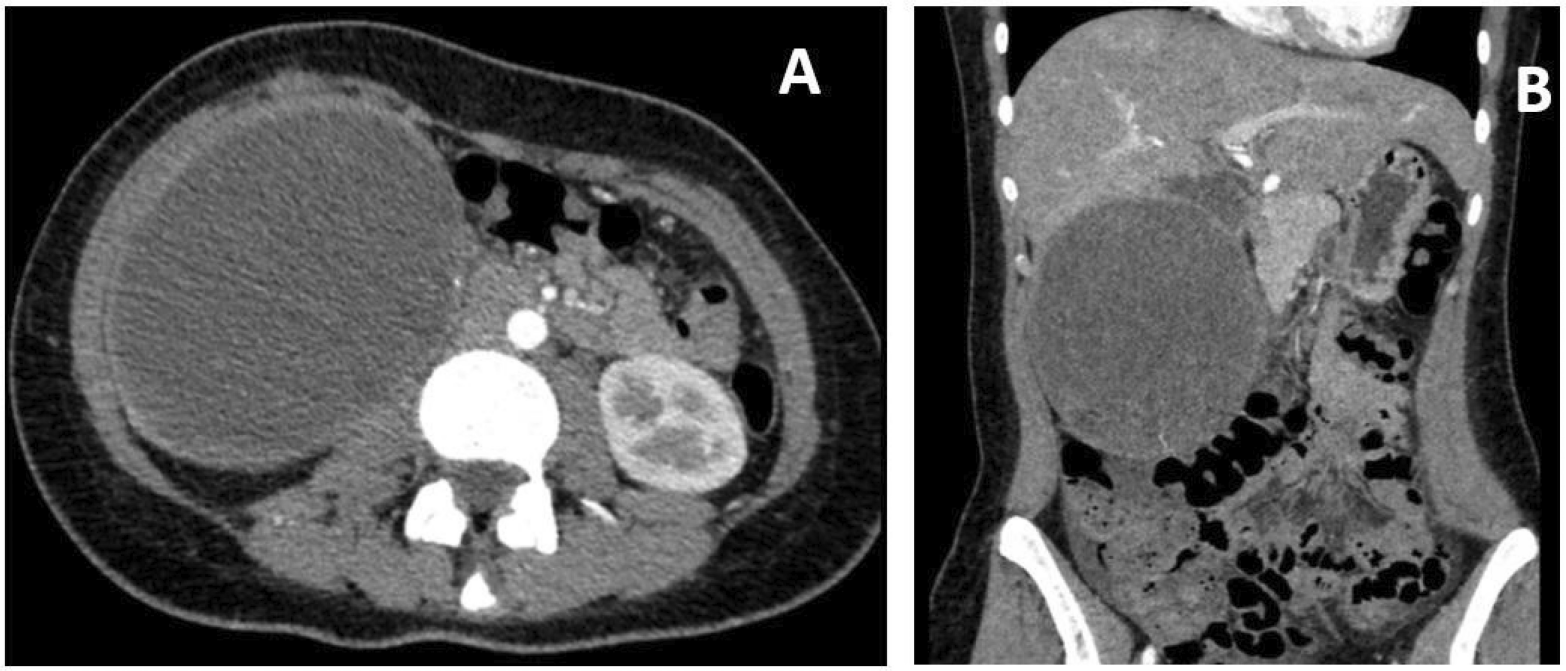
Contrast tomography of the abdomen. (A) Axial cut. (B) Coronal cut.

- Primary concerns and symptoms: Chronic abdominal pain in the right hypochondrium, nonsignificant weight loss.
- Relevant interventions and outcome: Percutaneous drainage of the lesion was unsuccessful. Ultrasound and abdominal tomography revealed a lesion with a heterogeneous solid and cystic appearance located in Segments 6 and 7 of the liver (Figure 1A,B), with no secondary findings elsewhere. Surgical resection was performed, and the postoperative period was uneventful.

Laboratory tests showed normal levels of CRP, bilirubin, LDH, AFP, Ca 19.9, CEA, and Ca125. The tumor was staged as R0 resection, indicating complete removal with no residual tumor.

**FIGURE 2:**
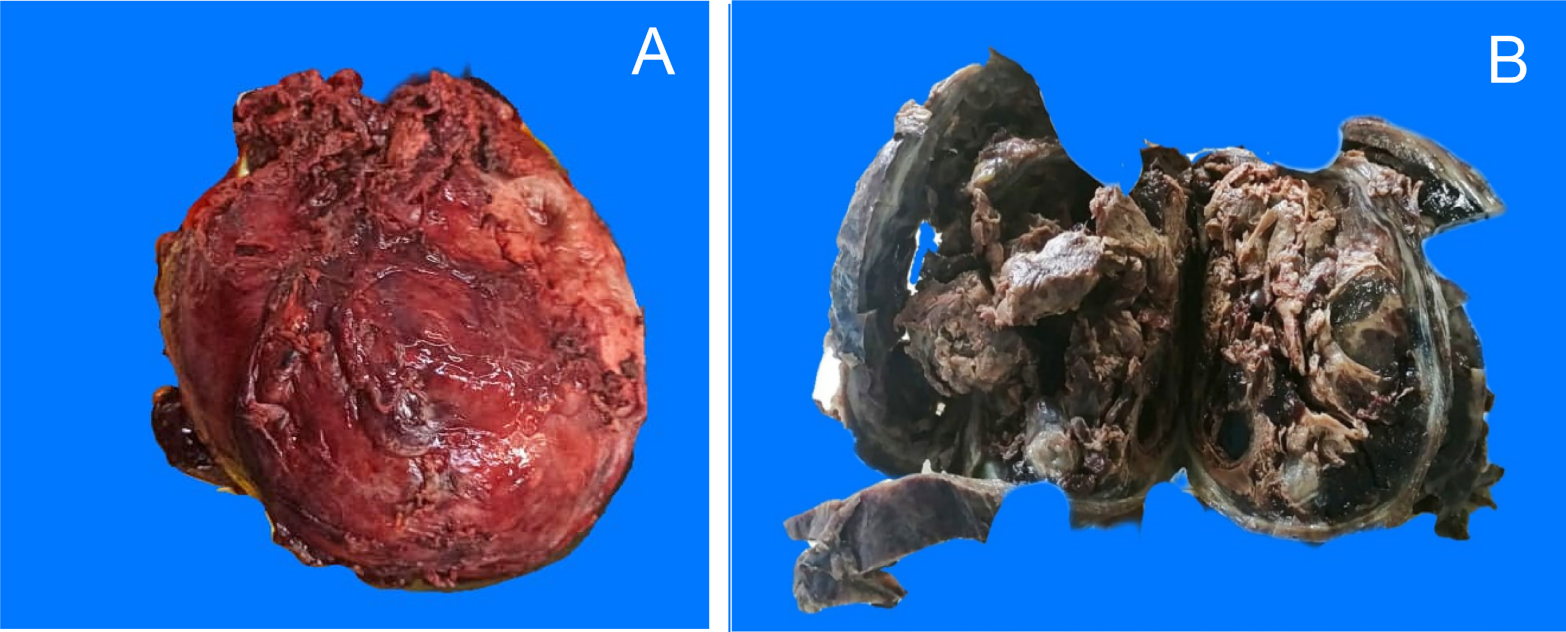
Macroscopic findings. (A) The fresh encapsulated tumor partially covered by liver tissue is identified, measuring 13 × 8 × 5 cm. (B) To the cut already fixed; the lesion is mostly cystic, with areas of necrosis, myxoid areas, and hemorrhage.

**FIGURE 3:**
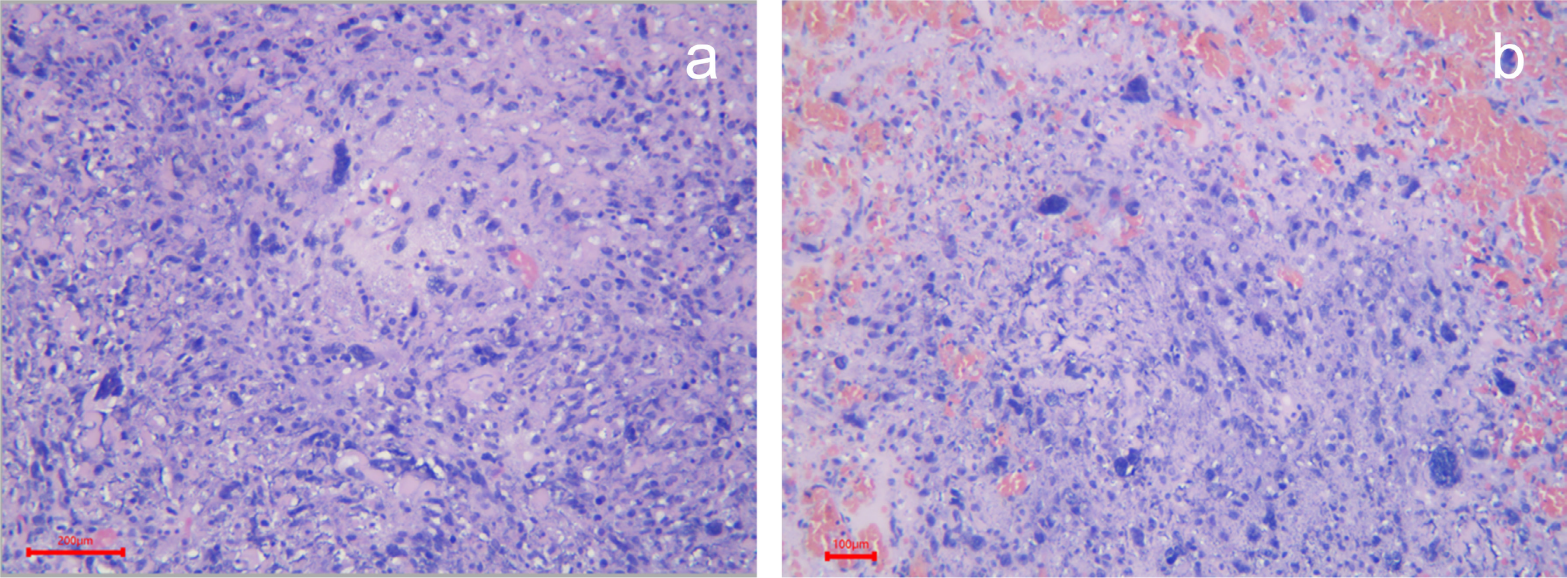
Histological findings with Hematoxylin–eosin (HE) staining. (A) 20× Scale bar 200 μm. Areas with round to spindle cell tumor cells and myxoid stroma. (B) 20× Scale bar 100 μm. There are pleomorphic cells with hyperchromatic nuclei.

**FIGURE 4:**
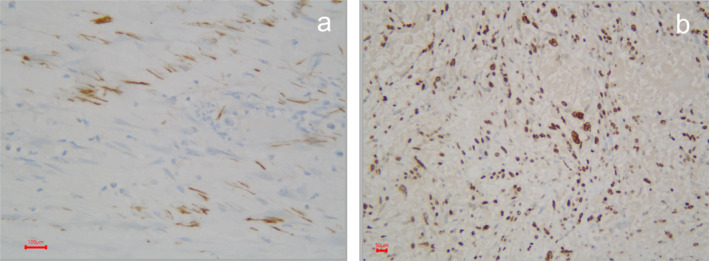
Immunohistochemical staining of tumor cells. (A) 40×. vimentin. It shows focal positivity in tumor cells in approximately 5% Scale bar 100 μm. (B) 20×. High Ki67 cell proliferation index (percentage of positive nuclei 50%) Scale bar 50 μm.

The tumor measured 13 × 8 × 5 cm, was encapsulated, and contained areas of necrosis (Figure 2A,B). Microscopically, it showed round tumor cells and a myxoid stroma, some pleomorphic cells with hyperchromatic nuclei (Figure 3A,B). Additionally, the immunohistochemical study was positive for vimentin, with a high Ki67 cell proliferation index (Figure 4A,B). She received adjuvant chemotherapy with Adriamycin and Ifosfamide, consisting of 6 cycles over 6 months. The patient has been followed for 34 months, and she remains free of disease.

## 2 Discussion

UESL is a mesenchymal tumor that occurs most frequently in children. Although few cases have been reported in adults, it is an aggressive disease, and early diagnosis is crucial given the possibility of surgical resection [9]. In some cases, the presentation can be confused with benign conditions such as biliary cystadenoma, bacterial infections like pyogenic abscesses, or parasitic infections such as hydatid cysts, especially when the cystic component of the lesion predominates, as in our case.

The presentation is usually nonspecific, with abdominal pain, a palpable mass in the abdomen, weight loss, and in some cases, fever. Tumor rupture or bleeding may be the initial clinical manifestation [10]. Imaging studies are essential in the approach to liver masses. In patients with UESL, a well‐defined, heterogeneous tumor with both solid and cystic components is characteristic [11]. There is no typical imaging pattern for this tumor, but discrepancies between imaging methods have been described. Ultrasound may show a predominantly solid component, while computed tomography and magnetic resonance imaging may reveal a predominantly cystic component [12].

Macroscopically, UESL has been described in the literature as large cystic tumors with areas of necrosis and hemorrhage alternating with cystic areas containing gelatinous material. The tumor may have infiltrative or circumscribed fibrous borders, which correlates with our case where a circumscribed fibrous border was observed. Microscopically, the tumor can display variable cellularity, with spindle‐shaped, stellate cells, anaplastic cells with high mitotic activity, and a stromal background with a myxoid appearance. Scattered bizarre, multinucleated, atypical cells may also be present, similar to our case (Figure 3). Tumor cells typically exhibit PAS‐D (periodic acid‐Schiff stain)‐positive hyaline eosinophilic globules in the cytoplasm, which may also be seen in the extracellular stroma. These hyaline globules can stain with α1‐antichymotrypsin (AAT) but not AFP, and their presence is a diagnostic feature. In our case, hyaline globules were not observed. Other immunohistochemical markers that may be focally positive and heterogeneous in tumor cells include desmin, CD68, vimentin, glypican‐3, and CD10, whereas myogenin, S‐100 protein, CD117, and HepPar 1 should be negative. Hematopoiesis is also commonly observed. Tumor growth toward the periphery may lead to plaques of trapped hepatocytes and dilated bile ducts [13, 14, 15, 16].

UESL is now considered curable with neoadjuvant chemotherapy in combination with delayed complete surgical resection [17, 18]. The current literature strongly supports the use of chemotherapy in conjunction with surgery to improve long‐term outcomes. Complete resection, when feasible, should always be pursued, as it significantly impacts survival, and chemotherapy helps to treat any potential residual microscopic disease.

## 3 Conclusion

It is important to consider UESL as a possible cause of liver masses in Central America and to undertake an early diagnostic approach that allows for multimodal management to improve disease‐free survival in these patients. UESL is a treatable disease with a favorable prognosis if diagnosed and managed appropriately. The key takeaway from this study is the critical role of a multidisciplinary approach in diagnosing and treating UESL, emphasizing the importance of considering this rare tumor in differential diagnoses.

## Author Contributions

**Amilcar Antonio Alfaro García:** writing – review and editing, conceptualization, methodology, writing – original draft, supervision, project administration. **Ludwing Bacon Fonseca:** investigation, validation, writing – review and editing, formal analysis. **Maria del Rosario Romero Alvarez:** writing – review and editing, software, data curation, visualization.

## Ethics Statement

Authors declare that the manuscript has been done in accordance with the Committee on Publication Ethics (COPE) guidelines and has been performed ethically and responsibly, with no falsification, plagiarism, image manipulation, or unethical research.

## Consent

Written informed consent was obtained from the patient for participation and publication.

## Conflicts of Interest

The authors declare no conflicts of interest.

## Data Availability

The data that support the findings of this study are available on request from the corresponding author. The data are not publicly available due to privacy or ethical restrictions.
